# Eccrine Porocarcinoma of the Chest Wall

**DOI:** 10.7759/cureus.26510

**Published:** 2022-07-02

**Authors:** Boris Kangabam, Ramthaipou Kamei

**Affiliations:** 1 Surgery, Jawaharlal Nehru Institute of Medical Sciences, Imphal, IND

**Keywords:** surgery, wide local excision, skin cancer, thorax, eccrine porocarcinoma

## Abstract

Eccrine porocarcinoma is a rare malignant tumor of skin sweat glands. It accounts for 0.005% of cutaneous tumors. It may present in a myriad of ways ranging from indolent swellings to aggressive tumors with metastasis. Notably, it is difficult to diagnose clinically. Histopathology is necessary and the pathologist often makes the diagnosis. Early intervention can lead to a curative outcome. We present a case of a 65-year-old man who presented with painless swelling in the upper back. Wide local excision was done with rhomboid flap reconstruction. The diagnosis was made postoperatively on histopathology examination. After 10 months of follow-up, there was no sign of recurrence.

## Introduction

Eccrine porocarcinoma is a rare malignant skin tumor that originates from sweat glands, representing 0.005% of epithelial cutaneous neoplasms. It was first described by Pinkus and Mehregan in 1963 [1]. Its presentation can be in a myriad of ways ranging from nodular swellings to ulcerations in the skin, often seen in the elderly [2]. It arises mostly on the lower limbs. Lymph node metastasis occurs in 20% of cases and distant metastasis in 10% of cases [3]. Notably, diagnosis is often delayed and can pose a challenge to the dermatologist, the surgeon, and the pathologist. Early recognition is crucial for a good outcome. Histopathology is necessary to make the diagnosis. Surgical resection is the mainstay of treatment [2]. Adjuvant treatment remains controversial [1]. Standard treatment guidelines are not defined owing to its rarity. Here, we present a 65-year-old man complaining of painless swelling in the upper back. Fine needle aspiration cytology (FNAC) initially reported features suggestive of soft tissue sarcoma. An incisional biopsy was done, which reported a scar with overlying bowenoid keratosis. The final diagnosis was made postoperatively with the aid of immunohistochemistry. Our case highlights the challenge clinically and histologically in making the elusive diagnosis preoperatively.

## Case presentation

**Figure 1:**
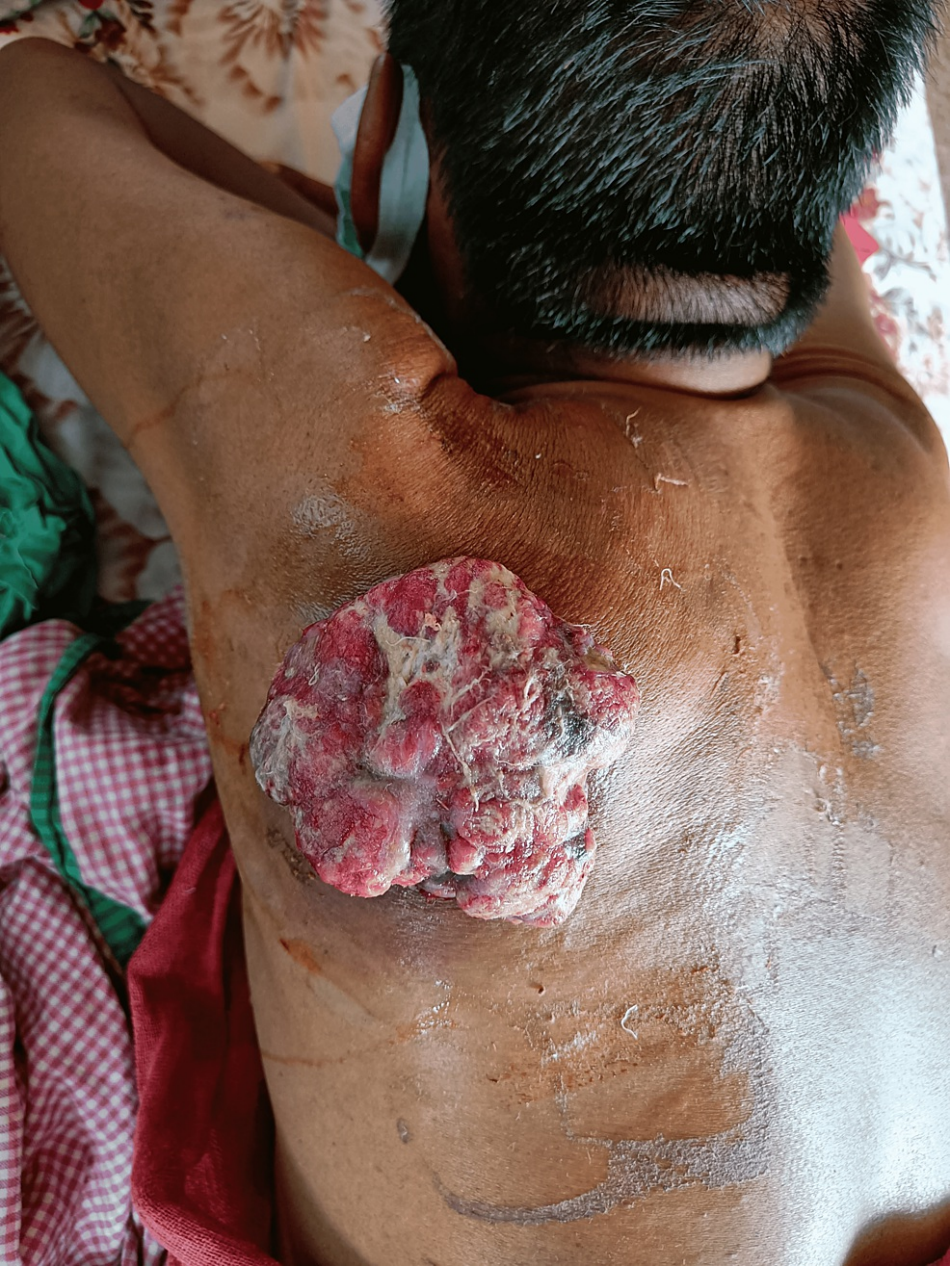
Reddish-colored ulceroproliferative growth on the left upper back.

A 65-year-old male farmer presented to the surgical outpatient department with complaints of swelling in the left upper back. It appeared two years back as a slow-growing, non-tender, reddish-colored swelling associated with pruritus intermittently. There was no history of discharge or trauma. He had no significant medical comorbidities and no history of cancer in the family. He underwent excision of the swelling two months back in another clinic but the excised specimen was not sent for biopsy. Since then, the swelling recurred and grew rapidly in size over the past two months. FNAC of the recurrent swelling was done, which showed features suggestive of soft tissue sarcoma, after which the patient did not attend follow-up. On clinical examination, there was a 7 x 8 x 4 cm reddish-colored ulceroproliferative firm swelling (Figure 1). It was firm, painless, and mobile. There were no neurovascular deficits and no restrictions on the mobility of the upper limbs. Clinically, lymph nodes were not palpable. There was no evidence of metastasis on systemic workup.

**Figure 2:**
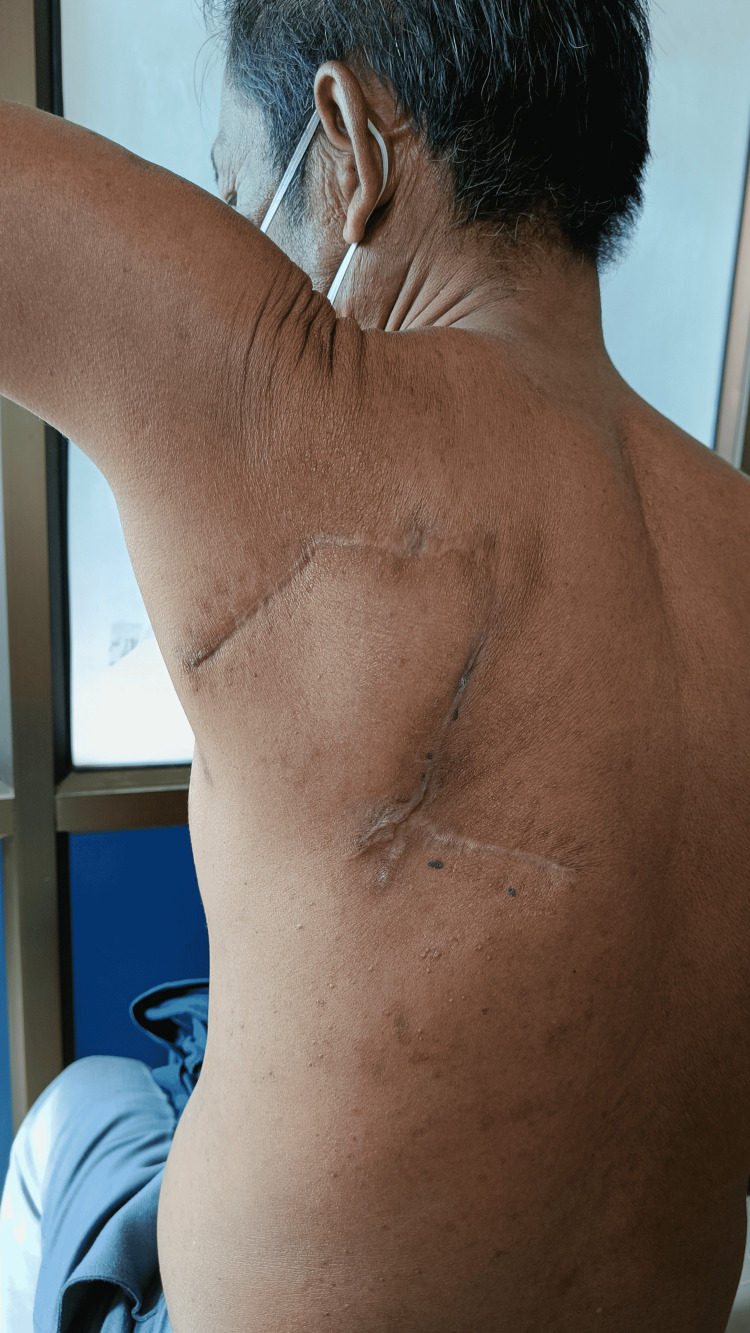
Healed operated site on follow-up after 10 months.

**Figure 3:**
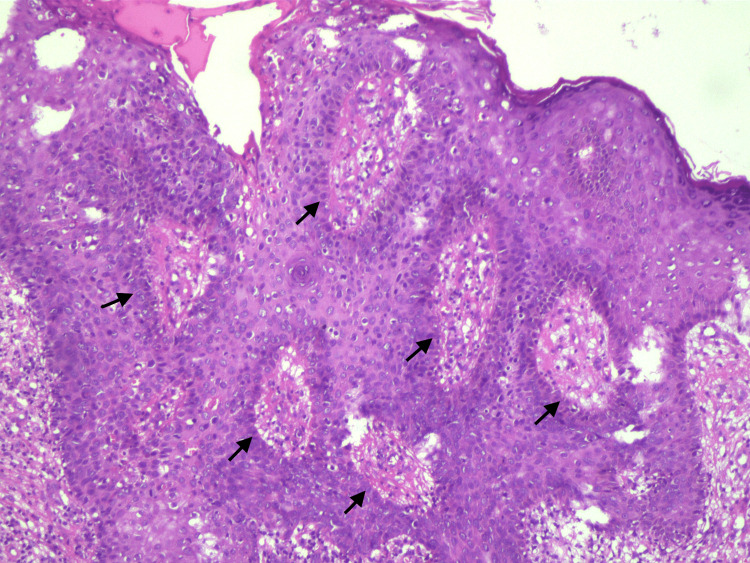
Histopathology of the swelling showing tumor composed of cells in the dermis in lobules (dark arrows) with broad pushing margins and interlacing cords (hematoxylin and eosin stain in 10x).

MRI of the chest with contrast identified an ovoid-shaped, T1 hypointense, T2 hyperintense lesion arising exophytically from the skin overlying the left upper back. It extended to the underlying subcutaneous tissue but there was no invasion of the underlying muscle. Incisional biopsy was done and it showed a scar with overlying bowenoid keratosis. However, immunohistochemistry was not done preoperatively. The patient underwent wide local excision of the swelling with at least a 1-cm margin on all sides followed by rhomboid mucocutaneous flap reconstruction. After an uneventful hospital stay, the patient was given antibiotics and analgesics and was discharged after five days. Arm stretching exercises were taught and the patient was advised not to lift heavy objects. He was advised to follow up with a medical oncologist for adjuvant therapy but the patient refused. He was regularly followed up on an outpatient basis every two months. After 10 months, the operative site appeared healthy (Figure 2). He had no medical complaints and there were no restrictions on the mobility of the upper limbs. There were no signs of recurrence on clinical and radiological assessment (Figure 2). Histopathological analysis was suggestive of eccrine porocarcinoma (Figure 3).

Surgical margins were clear. There was no evidence of lymphovascular or perineural invasion. On immunohistochemistry, it was positive for cytokeratin (AE1/AE3) and epithelial membrane antigen (EMA).

## Discussion

Eccrine porocarcinoma is a rare malignant skin tumor of sweat gland origin, accounting for 0.005% of epithelial cutaneous neoplasms [1]. It is known by many names like malignant hidroacanthoma simplex, malignant syringoacanthoma, and dysplastic poroma [3]. It is seen in the elderly mostly in the seventh and eighth decades of life. It has no gender predilection. The usual presentation is a slow-growing nodule associated with warty or ulcerative changes [4]. It may also present as rapidly growing multi-nodular swelling, which is seen in either metastatic or local recurrence after excision, as in this case.

Immunodeficiency, sunlight exposure, and eccrine poroma are reported to be risk factors for the formation of porocarcinoma. The pathogenesis is still not understood. Loss of TP53 gene heterozygosity and somatic mutation of cyclin-dependent kinase inhibitor 2A/B gene encoding for TP16 and TP14 have been found to be associated with it [5].

The diagnosis of porocarcinoma is often notoriously challenging based on clinical and radiological assessment. Radiologically, mushroom-like exophytic skin appendage, as seen in mycosis fungicides, could be suggestive of skin adnexal tumor. Histopathology with immunohistochemistry is crucial. A mitotic index of more than 14 mitotic cells per high power field, lymphovascular invasion, and a tumor depth of more than 7 mm are considered to carry a worse prognosis [1]. Cytokeratin (34BE12, AE1/AE3), p63, CAM 5.2 antigen, cytokeratin 7 (CK7), EMA, and carcinoembryonic antigen (CEA) may be done to support the diagnosis through immunohistochemistry but are not strictly necessary [6].

There are no proper guidelines for the treatment of this disease [1]. Surgical resection with clear margins may provide curative outcomes in 70-80% of cases [3]. Wide local excision and Mohs micrographic surgery are usually performed. Lymph node clearance is a matter of debate and has shown to have no impact on disease-free survival. Guidelines for adjuvant therapy are still lacking due to its rarity [1]. Radiotherapy, immunotherapy, and chemotherapy options are tailored accordingly in patients with metastasis and refractory cases [7].

## Conclusions

Eccrine porocarcinoma is a rare malignant tumor of the skin sweat gland. Due to its varied presentation, recognition of this tumor can be challenging. It is often diagnosed late in the course of the disease. A lack of a representative sample may lead to a false diagnosis. A biopsy is crucial and adequate tissue samples should be taken for histopathology. Immunohistochemistry can aid in making the correct diagnosis. Communication between the pathologist and the clinician is necessary. Early recognition and intervention may provide a curative outcome. Given its rarity and variations, clinical encounters and experiences need to be studied for better understanding.
